# Supplementary material for: Lanthanum Supplementation Alleviates Tomato Root Growth Suppression under Low Light Stress
Source: Plants (Basel). 2023 Jul 16;12(14):2663. doi: 10.3390/plants12142663 (PMC10384870; doi:10.3390/plants12142663)
Supplement: Supplementary file 1 [file plants-12-02663-s001.zip › plants-2492101-supplementary.pdf]

Table S1. Primers for qRT-PCR analysis of genes expressed in tomato roots under low light stress.

Gene name	Primers	Nucleotide sequences (5'→3')	Size of amplified gene (bp)
Superoxide dismutase	Solyc11g066390 F<br>Solyc11g066390 R	CGGTCCAAACTCAGTTGTTG<br>CAGCATTGCCAGTGGTAAGA	101
Ascorbate peroxidase	Solyc06g005150 F<br>Solyc06g005150 R	AGCCCATTAGGGAGCAGTTT<br>GAACATCAGGTCCTCCCGTAAC	96
Catalase	Solyc04g082460 F<br>Solyc04g082460 R	GGCTTGATCCTTGTCGTCAT<br>CGGTGCCCAAGATCTGTAAT	134
Glutathione peroxidase	Solyc09g064850 F<br>Solyc09g064850 R	TGGGAGCTTCCAAATCAGTT<br>TGTGCTTGTGAACCCACATT	146
Chalcone flavanone isomerase	Solyc02g083890 F<br>Solyc02g083890 R	TGGGAAACAATCAGCATTGA<br>CCAAATTCCCCATAGCAAGA	71
MYB transcription factor	Solyc06g075660 F<br>Solyc06g075660 R	ACCAGGGCGAACTGATAATG<br>TGGCAGAGGAAGAGGAACAA	134
Phenylalanine ammonia lyase	Solyc05g056170 F<br>Solyc05g056170 R	TACATTGCCGGCCTTTTAAC<br>AATACCAGCCTGTTGGAACG	99
Trans-cinnamate 4 hydroxylase	Solyc05g047530 F<br>Solyc05g047530 R	GTTGGCTAACAACCCTGCAT<br>ACCTTACCACCAGCAACAGC	105
Gibberellin 2-oxidase	Solyc01g079200 F<br>Solyc01g079200 R	TTCGTAGGGGACTCCTTTCAG<br>TTGACACCACTCTATGTCTTACAC	70
Abscisic acid stress ripening protein	Solyc04g071590 F<br>Solyc04g071590 R	AGCACCATAGCCATCTCCAG<br>GGTCTTTCTTTGCCTTGTGC	93
Ethylene-responsive transcription factor	Solyc04g071770 F<br>Solyc04g071770 R	TGGAGAATCATCTTCCTCTGTTA<br>CGATATGCTGCTCGTTTGTG	73
EIN3-binding F-box protein	Solyc12g009560 F<br>Solyc12g009560 R	AACCTTAGCGACTGCCTGAA<br>CATCGGTAACCTTCCTGCAT	112
Cytokinin riboside 5'-monophosphate phosphoribohydrolase	Solyc08g062820 F<br>Solyc08g062820 R	TGGTGGAAGTGTTGGATTGA<br>TCATGCATGTCCGAAACAAT	150
Heavy metal-associated isoprenylated plant protein 26	Solyc11g007200 F<br>Solyc11g007200 R	TGTCATGCCAAGGTTGTGTT<br>TTCCGGTTCCACATTTCCTA	122
Heavy metal-associated isoprenylated plant protein 3	Solyc09g008200 F<br>Solyc09g008200 R	GGAGACAAAGTCCGAAGCGA<br>CACAGGCATCGCGTAATGTG	73
NAC domain	Solyc03g097650 F<br>Solyc03g097650 R	TTCTTGTCCCTTGCCTGCTT<br>TCACCAGGCAAATTCCAGGG	75
Clathrin light chain	Solyc03g006510 F<br>Solyc03g006510 R	AGGCTTACGATCTCGGTGAA<br>TCAAGACGGACAGCATTTTG	133
Anaphase promoting complex subunit 13	Solyc08g006570 F<br>Solyc08g006570 R	TCCCGTTACCACCCGTACTAC<br>AGGTCGTGCCATGTATCTCC	94
Auxin responsive SAUR protein	Solyc12g089270 F<br>Solyc12g089270 R	GGGCCCCAATTACAAAAGAT<br>GGAATGACTAATGGGCCAGAG	123
Small auxin up-regulated RNA6	Solyc01g110590 F<br>Solyc01g110590 R	GCCTTCCAATTGACGTACC<br>CGAACCCAAATTCTTCCTCA	141
Phytochrome-interacting factor 4 (PIF4)	Solyc07g043580 F<br>Solyc07g043580 R	ATGAAGGCCTTGCAAGAGCT<br>GCCACTTCCCATCCACATCA	126
Phytochrome-interacting factor 7 (PIF7)	Solyc03g115540 F<br>Solyc03g115540 R	TTCTGTTAGCCCTGCCAGTG<br>GCCGATACAAAGCTGCCATG	131
Transcription factor Nozzle	Solyc07g063670 F<br>Solyc07g063670 R	CTTACTGCCTCTTCCCCACC<br>CTCCCGTTGAGGTCTGGTTC	144
Photosystem II reaction center W protein	Solyc09g065910 F<br>Solyc09g065910 R	CATGTCAAGCCCAGTAGCAAT<br>TCCAAATGGTAGCCCTGTTC	73
Chlorophyll *a-b* binding protein	Solyc06g063370 F<br>Solyc06g063370 R	CCGAGGGTCTATTGGACAGA<br>ATGCCTGATATTTGGCGAAG	123
Ribulose bisphosphate carboxylase	Solyc10g086580 F<br>Solyc10g086580 R	AGTGGAAATGCAGGAGAGCC<br>CTACCAGCTCCTGCATCGAG	122
Extensin-like protein	Solyc02g030220 F<br>Solyc02g030220 R	GCTTTGGAAATTTGGGACAAAG<br>AGCAACAACAGTGCTGGCTAC	77
Cellulose synthase	Solyc01g087210 F<br>Solyc01g087210 R	CTGGTGGTGCAAAACACATC<br>CTCCCTTACTGGGTCCACAAC	89
Pectin lyase	Solyc12g019120 F<br>Solyc12g019120 R	TGGGGAATTGGTAATTCTGG<br>AGCCCAAAAACCATCTCCTT	102
Polygalacturonase	Solyc03g116500 F<br>Solyc03g116500 R	AGATGGTATGGGCCAGGAGT<br>AAGCCGTTGGAGCATGATGA	80
Calcium-binding EF hand protein	Solyc10g044700 F<br>Solyc10g044700 R	TGTTGATGGTCCAATGATGG<br>TCAAAATGTTTGTCCGTCCA	75
Calcium binding protein CML 38	Solyc06g073830 F<br>Solyc06g073830 R	GCGATCAATTGGAGAGGGTA<br>CGTCGTTAAATCTCCGCCTA	110
Calcium-dependent lipid-binding protein	Solyc09g009370 F<br>Solyc09g009370 R	GAGCAGACAGATTGGTGCAA<br>AAAACAGCATGACACCACCA	122
Phosphate transporter	Solyc03g005555 F<br>Solyc03g005555 R	AGCGAGGAAGCCAAAATCGA<br>GCCGTCGAACAAATTCCCAC	82
Potassium transporter	Solyc02g087000 F<br>Solyc02g087000 R	GCATTACGGAACCCTGAAGA<br>TACCCAGGTTGGGACTCAAG	89
GAPDH	Solyc05g014470 F<br>Solyc05g014470 R	AAGCTGGTGCTGACTTCGTTG<br>CGTTGACACCCACAACAAAC	144
